# Synthesis and Antifungal Activity of Pyrimidine Derivatives Containing an Amide Moiety

**DOI:** 10.3389/fchem.2021.695628

**Published:** 2021-07-12

**Authors:** Wenneng Wu, Wenjun Lan, Chenyan Wu, Qiang Fei

**Affiliations:** Food and Pharmaceutical Engineering Institute, Guiyang University, Guiyang, China

**Keywords:** pyrimidine, amide, synthesize, antifungal activity, kiwifruit soft rot disease

## Abstract

In this study, 17 novel pyrimidine derivatives containing an amide moiety were synthesized. Then their *in vitro* antifungal activities against *Botryosphaeria dothidea* (*B. dothidea*), *Phomopsis* sp., and *Botrytis cinereal* (*B. cinereal*) were determined. A preliminary biological test showed that compounds 5-bromo-2-fluoro*-N*-(2-((2-methyl-6-(trifluoromethyl)pyrimidin-4-yl)oxy)phenyl)benzamide (**5f**) and 5-bromo-2-fluoro-*N*-(3-((2-methyl-6-(trifluoromethyl)pyrimidin-4-yl)oxy)phenyl)benzamide (**5o**) exhibited higher antifungal activity against *Phomopsis* sp., with an inhibition rate of 100% compared to that of Pyrimethanil at 85.1%. In particular, compound **5o** exhibited excellent antifungal activity against *Phompsis* sp., with the EC_50_ value of 10.5 μg/ml, which was even better than that of Pyrimethanil (32.1 μg/ml). As far as we know, this is the first report on the antifungal activities against *B. dothidea*, *Phomopsis* sp., and *B. cinereal* of this series of pyrimidine derivatives containing an amide moiety.

## Introduction

Plant fungal diseases pose serious threats to crop production and caused huge economic losses throughout the world (Strange and Scott, 2005). In recent years, crop cultivators continually battle with plant fungal diseases affecting crops. The available traditional fungicides used for plant fungal diseases control represent a danger to the living system by killing not only the target fungi but also affecting beneficial living systems (Patel et al., 2014). To protect crops from fungal diseases, commercial agriculture relies heavily on the inputs of chemical pesticides. The resistance of plant fungal diseases against fungicides is rapidly becoming a serious problem. Therefore, the development of novel and promising fungicides is urgently required.

**FIGURE 1:**
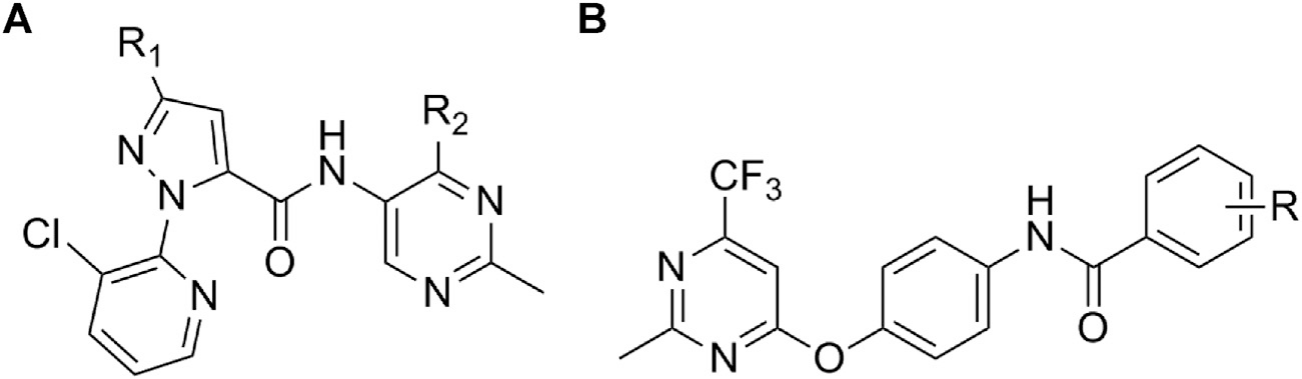
Structures of pyrimidine derivatives containing an amide moiety reported in our previous works.

Pyrimidines are important substances in the synthesis of various active molecules that are extensively used in the intermediate skeleton of agrochemicals (Chen et al., 2015; Wu et al., 2015) and have attracted more and more attention due to their extensive biological activities (Sun et al., 2006), including antiviral (Wu et al., 2016a), antibacterial (Wu et al., 2016b), antifungal (Wu et al., 2019, 2020), and insecticidal (Liu et al., 2017; Wu et al., 2020) activities. For example, Zan et al. reported pyrimidine derivatives bearing a dithioacetal moiety as effective antiviral agents for controlling the tomato chlorosis virus (ToCV) (Zan et al., 2021). Zhang et al. found a series of arylpyrazole pyrimidine ether derivatives with promising bioactivity for combating cucumber downy mildew (Zhang et al., 2019). Li and coworkers showed that pyrimidine thiourea derivatives had good herbicidal activities against *Digitaria adscendens* and *Amaranthus retroflexus* (Li et al., 2021a). In the past few years, several pyrimidine compounds have been commercialized as fungicides (such as Pyrimethanil, Fenarimol, Diflumetorim, and Mepanioyrim) for controlling plant fungal diseases, such as cucumber gray mold (Wang et al., 2021), grape downy mildew (Huang et al., 2018), kiwifruit leaf spot (Shi et al., 2021), and so on. Due to the excellent features of low toxicity, the fact that it is easily synthetized and derived, and considering pyrimidine as a parent compound, the development of promising agrochemical candidates will soon become a reality. Meanwhile, amine, a key moiety in heterocyclic chemistry, play a leading role in pesticide chemistry due to their potent bioactivities including antifungal (Cheng et al., 2020; Chen et al., 2021a), antibacterial (Chen et al., 2021a), antiviral (Tang et al., 2020), herbicidal (Sun et al., 2020), and insecticidal (Chen et al., 2021b; Li et al., 2021b) activities. In our previous work, we reported a series of novel pyrimidine derivatives containing an amine moiety (Figure 1) and found that the target compounds revealed certain antifungal, insecticidal, and antiviral activities (Wu et al., 2016a: Wu et al., 2020).

This study aimed to design and synthesize a series of novel pyrimidine derivatives containing an amide moiety, and investigate their *in vitro* antifungal activities against *Botryosphaeria dothidea* (*B. dothidea*), *Phomopsis* sp., and *Botrytis cinereal* (*B. cinereal*). A biological test showed that compound 5-bromo-2-fluoro-*N*-(3-((2-methyl-6-(trifluoromethyl)pyrimidin-4-yl)oxy)phenyl)benzamide (**5o**) exhibited excellent antifungal activity against *Phompsis* sp., with an EC_50_ value of 10.5 μg/ml, which is even better than Pyrimethanil (32.1 μg/ml).

## Materials and Methods

### General Information

The melting points of the products were determined on an XT-4 binocular microscope (Beijing Tech Instrument Co., China) and were not corrected. ^1^H and ^13^C NMR (solvent DMSO-*d*_*6*_) spectra were recorded on a Bruker AVANCE HD 600 MHz Digital NMR Spectrometer (Bruker Company, Billerica, MA, United States) at room temperature using TMS as an internal standard. High-resolution mass spectrometry (HRMS) was carried out on an Agilent Technologies 6540 UHD Accurate-Mass Q-TOF LC/MS (Agilent Technologies, Palo Alto, CA, United States). All anhydrous solvents were dried and purified according to standard techniques before use. Unless otherwise noted, all common reagents and solvents were used as obtained from commercial supplies without further purifications.

### Synthesis

#### General Procedure for the Preparation of the Intermediates 2–4

**SCHEME 1:**
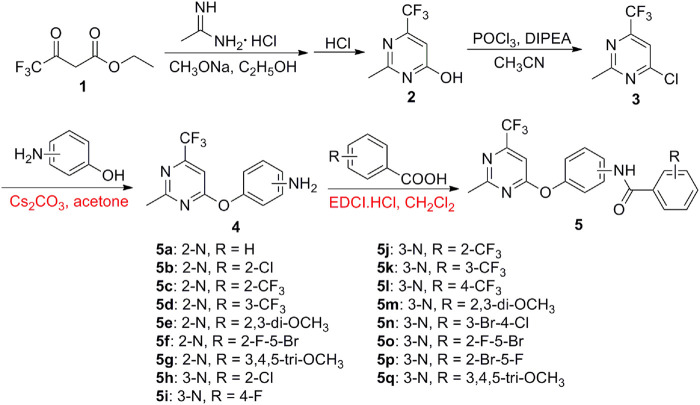
Synthetic routes of the target compounds 5a–5q.

As shown in Scheme 1, to a 250 ml round bottom flask, trifluoroacetoacetate (**1**, 0.05 mol), acetamidine hydrochloride (0.05 mol), sodium methoxide (0.075 mol), and ethanol (100 ml) were added and refluxed for 10 h. After that, the mixture was acidified with dilute HCl to pH 7. The crude products were extracted using ethyl acetate to produce intermediate **2**. Then, intermediate **2** (0.05 mol), POCl_3_ (0.1 mol), and CH_3_CN (120 ml) were added to a 250 ml round bottom flask to react for 0.5 h at a reflux temperature and then diisopropylethylamine (0.06 mol) was added dropwise. After continuously refluxing for 8 h, excess POCl_3_ and CH_3_CN were distilled and then ice water (60 ml) was added. Finally, the mixture was alkalified with dilute NaOH to pH 9 and extracted using CH_2_Cl_2_ to give intermediate **3**.

To a 50 ml three-necked round-bottomed flask equipped with a magnetic stirrer, the key intermediate **3** (0.01 mol) was dissolved in acetone (50 ml), Cs_2_CO_3_ (0.012 mol), and 3-aminophenol or 2-aminophenol (0.01 mol) were added. The reactions reacted for 5–6 h at room temperature, and the solvent was removed. The residue was added with water, the precipitate formed was filtered off and recrystallized from ethanol to give the intermediate **4**.

#### General Procedure for the Preparation of the Target Compounds **5a-5q**

To a 50 ml three-necked round-bottomed flask equipped with a magnetic stirrer, the key intermediate **4** (0.02 mol), aromatic acid (0.02 mol), and dimethylaminopyridine (0.0002 mol) dissolved in dichloromethane (10 ml), and 1-(3-dimethylaminopropyl)-3-ethylcarbodiimide hydrochloride (0.03 mol) were added. The reactions were reacted for 8–10 h at room temperature. The solvent was then dried under vacuum and recrystallized from ethanol to give the pure target compounds **5a–5q**.

The structures were confirmed by ^1^H NMR, ^13^C NMR, and HRMS. ^1^H NMR, ^13^C NMR, and HRMS spectral data for the target compounds **5a–5q** are reported in the Supplementary Material. In the ^1^H NMR spectra of **5a–5q**, one singlet at δ 9.70–10.74 ppm were attributed to the -CONH-. A singlet at δ 6.90–7.54 ppm demonstrated the presence of the proton of the pyrimidine fragment. A singlet at δ 2.31–2.54 ppm integrating for three protons was assigned to Pyrimidine-CH_3_ protons. The structure of **5a–5q** was also confirmed by its HRMS spectral data. In all HRMS spectrum, the molecular ion peak was noticed m/z for ([M + H]^+^) corresponding to all of the target molecular weight.

***N*-(2-((2-methyl-6-(trifluoromethyl)pyrimidin-4-yl)oxy)phenyl)benzamide** (**5a**). White solid; yield 75%; m.p. 173–175°C; ^1^H NMR (DMSO-*d*_6_, 600 MHz, ppm) *δ*: 9.72 (s, 1H), 7.96 (d, 2H, *J* = 7.8 Hz), 7.76 (d, 1H, *J* = 7.2 Hz), 7.71 (t, 1H, *J* = 8.4 Hz), 7.53 (t, 2H, *J* = 9.0 Hz), 7.41–7.31 (m, 3H, Ph-H), 6.90 (s, 1H), 2.32 (s, 3H); ^13^C NMR (DMSO-*d*_6,_ 150 MHz, ppm) *δ*: 168.66, 164.39, 162.11, 152.99 (q, *J* = 33.6 Hz), 144.29, 134.43, 130.75, 130.08, 129.35, 129.22, 126.84, 126.40, 125.99, 124.06, 122.24 (q, *J* = 272.85 Hz), 100.17, 25.75; HRMS (calcd.) for C_19_H_14_F_3_N_3_O_2_ (M + H)^+^ 374.1107, found 374.1107.

**2-Chloro-*N*-(2-((2-methyl-6-(trifluoromethyl)pyrimidin-4-yl)oxy)phenyl)benzamide** (**5b**). White solid; yield 49%; m.p. 157–158°C; ^1^H NMR (DMSO-*d*_6_, 600 MHz, ppm) δ: 9.73 (s, 1H), 7.93 (d, 1H, *J* = 7.8 Hz), 7.82 (d, 1H, *J* = 6.0 Hz), 7.66–7.63 (m, 2H), 7.49–7.46 (m, 1H), 7.43 (d, 1H, *J* = 7.8 Hz), 7.40–7.33 (m, 2H), 6.91 (s, 1H), 2.38 (s, 3H); ^13^C NMR (DMSO-*d*_6,_ 150 MHz, ppm) δ: 168.75, 162.96, 162.24, 153.25 (q, *J* = 33.6 Hz), 144.14, 134.62, 133.51, 132.34, 131.70, 130.75, 128.70, 127.80, 127.14, 126.60, 123.97, 122.28 (q, *J* = 272.55 Hz), 120.46, 100.24, 25.86; HRMS (calcd.) for C_19_H_13_ClF_3_N_3_O_2_ (M + H)^+^ 408.0721, found 408.0720.

***N-*(2-((2-methyl-6-(trifluoromethyl)pyrimidin-4-yl)oxy)phenyl)-2-(trifluoromethyl)benzamide** (**5c**). White solid; yield 46%; m.p. 148–150°C; ^1^H NMR (DMSO-*d*_6_, 600 MHz, ppm) δ: 9.77 (s, 1H), 7.94 (t, 2H, *J* = 6.0 Hz), 7.86–7.81 (m, 3H), 7.41–7.36 (m, 3H), 6.92 (s, 1H), 2.39 (s, 3H); ^13^C NMR (DMSO-*d*_6,_ 150 MHz, ppm) δ: 168.75, 163.97, 162.25, 153.52 (q, *J* = 33.6 Hz), 144.03, 133.33, 133.21, 131.22, 130.77, 129.50, 128.33, 128.11 (q, *J* = 31.95 Hz), 127.52, 127.36, 126.57, 124.65 (q, *J* = 272.1 Hz), 123.45, 122.25 (q, *J* = 272.7 Hz), 120.45, 100.23, 25.85; HRMS (calcd.) for C_20_H_13_F_6_N_3_O_2_ (M + H)^+^ 442.0985, found 442.0979.

***N-*(2-((2-methyl-6-(trifluoromethyl)pyrimidin-4-yl)oxy)phenyl)-3-(trifluoromethyl)benzamide** (**5d**). White solid; yield 68%; m.p. 138–140°C; _1_H NMR (DMSO-*d*_6_, 600 MHz, ppm) δ: 9.81 (s, 1H), 8.30 (d, 1H, *J* = 7.8 Hz), 8.14 (s, 1H), 8.09 (d, 1H, *J* = 7.8 Hz), 7.82 (t, 1H, *J* = 7.8 Hz), 7.78 (d, 1H, *J* = 7.2 Hz), 7.48 (d, 1H, *J* = 8.4 Hz), 7.41–7.35 (m, 2H), 6.91 (s, 1H), 2.30 (s, 3H); ^13^C NMR (DMSO-*d*_6,_ 150 MHz, ppm) δ: 168.66, 163.13, 162.04, 153.33 (q, *J* = 34.2 Hz), 144.11, 134.06, 130.82, 130.77, 130.60, 130.55, 130.16 (q, *J* = 31.65 Hz), 127.09, 126.44, 126.08, 126.00, 124.92 (q, *J* = 270.9 Hz), 124.18, 123.10 (q, *J* = 270.6 Hz), 100.10, 25.60; HRMS (calcd.) for C_20_H_13_F_6_N_3_O_2_ (M + H)^+^ 442.0985, found 442.0979.

**2,3-Dimethoxy*-N*-(2-((2-methyl-6-(trifluoromethyl)pyrimidin-4-yl)oxy)phenyl)benzamide** (**5e**). White solid; yield 50%; m.p. 130–131°C; ^1^H NMR (DMSO-*d*_6_, 600 MHz, ppm) δ: 7.63 (s, 1H),7.54 (d, 1H, *J* = 7.6 Hz), 7.46 (t, 1H, *J* = 7.4 Hz), 7.41 (d, 1H, *J* = 8.1 Hz), 7.35–7.33 (m, 2H), 7.06 (d, 1H, *J* = 8.1 Hz), 6.97 (t, 1H, *J* = 7.9 Hz), 6.92 (d, 1H, *J* = 7.6 Hz), 3.76 (s, 3H), 3.69 (s, 3H), 2.40 (s, 3H); ^13^C NMR (DMSO-*d*_6,_ 150 MHz, ppm) δ: 169.52, 168.91, 162.84, 156.21 (q, *J* = 35.25 Hz), 152.54, 147.60, 145.80, 132.19, 131.54, 130.54, 127.24, 124.32, 124.10, 121.66 (q, *J* = 273.45 Hz), 115.48, 108.51, 103.05, 61.44, 56.28, 25.56; HRMS (calcd.) for C_21_H_18_F_3_N_3_O_4_ (M + H)^+^ 434.1322, found 434.1318.

**3,4,5-trimethoxy*-N*-(2-((2-methyl-6-(trifluoromethyl)pyrimidin-4-yl)oxy)phenyl)benzamide** (**5g**). White solid; yield 58%; m.p. 127–128°C; ^1^H NMR (DMSO-*d*_6_, 600 MHz, ppm) δ: 9.74 (s, 1H), 7.72 (d, 1H, *J* = 7.8 Hz), 7.40–7.35 (m, 3H),7.21 (s, 2H, Ph-H), 6.89 (s, 1H),3.78 (s, 6H), 3.75 (s, 3H) 2.33 (s, 3H); ^13^C NMR (DMSO-*d*_6,_ 150 MHz, ppm) δ: 168.77, 163.92, 153.21 (q, *J* = 34.6 Hz), 153.18, 142.77, 130.66, 126.93, 124.22, 120.40 (q, *J* = 272.3 Hz),107.36, 60.67, 56.37, 25.76; HRMS (calcd.) for C_22_H_20_F_3_N_3_O_5_ (M + H)^+^ 464.1428, found 464.1420.

**2-Chloro-*N*-(3-((2-methyl-6-(trifluoromethyl)pyrimidin-4-yl)oxy)phenyl)benzamide** (**5h**). White solid; yield 62%; m.p.136–139°C; 1H NMR (DMSO-*d*_*6*_, 600 MHz, ppm) δ: 10.74 (s, 1H), 7.75 (s, 1H), 7.62–7.58 (m, 3H), 7.54–7.52 (m, 2H), 7.49–7.45 (m, 2H), 7.04 (dd, 1H, *J*_1_ = 1.3 Hz, *J*_2_ = 8.0 Hz), 2.54 (s, 3H); ^13^C NMR (DMSO-*d*_6,_ 150 MHz, ppm) δ: 170.52, 169.76, 165.62, 156.54 (q, *J* = 34.65 Hz), 155.83, 152.36, 140.85, 137.16, 131.74, 130.56, 130.40, 130.18, 129.43, 127.78, 123.67 (q, *J* = 273.45 Hz), 117.52, 117.46, 112.91, 103.46, 25.94; HRMS (calcd.) for C_19_H_13_ClF_3_N_3_O_2_ (M + H)^+^ 408.0721, found 408.0716.

**4-Fluoro*-N*-(3-((2-methyl-6-(trifluoromethyl)pyrimidin-4-yl)oxy)phenyl)benzamide** (**5i**). White solid; yield 85%; m.p. 136–139°C; ^1^H NMR (DMSO-*d*_6_, 600 MHz, ppm) δ: 9.72 (s, 1H), 8.06–8.04 (dd, 2H, *J*_1_ = 5.4 Hz, *J*_2_ = 8.4 Hz), 7.79 (d, 1H, *J* = 7.2 Hz), 7.41–7.35 (m, 5H), 7.31 (t, 1H, *J* = 7.8 Hz), 6.91 (s, 1H), 2.37 (s, 3H); ^13^C NMR (DMSO-*d*_6,_ 150 MHz, ppm) δ: 168.67, 167.65, 165.07, 163.46, 162.07, 153.20 (q, *J* = 33.75 Hz), 144.08, 133.11, 130.74, 126.87, 126.30, 125.99, 125.89, 124.18, 122.23 (q, *J* = 272.85 Hz), 116.48, 100.25, 25.75; HRMS (calcd.) for C_19_H_13_F_4_N_3_O_2_ (M + H)^+^ 392.1017, found 392.1013.

***N-*(3-((2-methyl-6-(trifluoromethyl)pyrimidin-4-yl)oxy)phenyl)-2-(trifluoromethyl)benzamide** (**5j**). White solid; yield 73%; m.p. 140–142°C; ^1^H NMR (DMSO-*d*_6_, 600 MHz, ppm) δ: 10.80 (s, 1H), 7.87 (d, 1H, *J* = 7.7 Hz), 7.82 (t, 1H, *J* = 7.3 Hz), 7.75–7.72 (m, 3H), 7.58 (d, 1H, *J* = 7.9 Hz), 7.52 (s, 1H), 7.47 (t, 1H, *J* = 8.2 Hz), 7.05 (d, 1H, *J* = 7.7 Hz), 2.54 (s, 3H); ^13^C NMR (DMSO-*d*_6,_ 150 MHz, ppm) δ: 170.48, 169.75, 166.26, 156.33 (q, *J* = 33.6 Hz), 152.36, 140.81, 136.36, 133.14, 130.55, 129.02, 126.84, 126.46 (q, *J* = 31.05 Hz), 123.32 (q, *J* = 271.8 Hz), 121.84 (q, *J* = 272.85 Hz), 117.50, 112.97, 103.46, 25.91; HRMS (calcd.) for C_20_H_13_F_6_N_3_O_2_ (M + H)^+^ 442.0985, found 442.0982.

***N-*(3-((2-methyl-6-(trifluoromethyl)pyrimidin-4-yl)oxy)phenyl)-3-(trifluoromethyl)benzamide** (**5k**). White solid; yield 73%; m.p. 101–103°C; ^1^H NMR (DMSO-*d*_6_, 600 MHz, ppm) δ: 10.65 (s, 1H), 8.30 (s, 1H), 8.27 (d, 1H, *J* = 7.8 Hz), 7.97 (d, 1H, *J* = 7.7 Hz), 7.81–7.79 (m, 2H), 7.71 (d, 1H, *J* = 8.1 Hz), 7.50 (s, 1H), 7.48 (d, 1H, *J* = 8.2 Hz), 7.05 (d, 1H, *J* = 8.0 Hz), 2.53 (s, 3H); ^13^C NMR (DMSO-*d*_6,_ 150 MHz, ppm) δ: 170.56, 169.79, 164.71, 156.34 (q, *J* = 33.6 Hz), 152.29, 140.81, 136.01, 132.35, 130.22, 130.02, 129.81 (q, *J* = 31.8 Hz), 128.74, 125.31 (q, *J* = 271.8 Hz), 124.75, 123.52, 121.82 (q, *J* = 273.0 Hz), 118.39, 117.52, 113.84, 103.36, 25.91; HRMS (calcd.) for C_20_H_13_F_6_N_3_O_2_ (M + H)^+^ 442.0985, found 442.0983.

***N-*(3-((2-methyl-6-(trifluoromethyl)pyrimidin-4-yl)oxy)phenyl)-4-(trifluoromethyl)benzamide** (**5l**). White solid; yield 80%; m.p. 94–96°C; ^1^H NMR (DMSO-*d*_6_, 600 MHz, ppm) δ: 10.66 (s, 1H), 8.15 (d, 2H, *J* = 8.4 Hz), 7.94 (d, 2H, *J* = 7.8 Hz), 7.79 (t, 1H, *J* = 1.8 Hz), 7.69 (d, 1H, *J* = 9.6 Hz), 7.52 (s, 1H), 7.49 (t, 1H, *J* = 8.4 Hz), 7.06 (d, 2H, *J* = 7.8 Hz), 2.53 (s, 3H); ^13^C NMR (DMSO-*d*_6,_ 150 MHz, ppm) δ: 170.59, 169.80, 165.13, 156.34 (q, *J* = 34.6 Hz), 152.31, 140.83, 138.99, 132.11 (q, *J* = 31.62 Hz),130.52, 129.16, 125.98, 125.31(q, *J* = 273.25 Hz), 120.05 (q, *J* = 272.51 Hz), 118.35, 117.61 103.48, 25.98; HRMS (calcd.) for C_20_H_13_F_6_N_3_O_2_ (M + H)^+^ 442.0985, found 442.0978.

**2,3-Dimethoxy*-N*-(3-((2-methyl-6-(trifluoromethyl)pyrimidin-4-yl)oxy)phenyl)benzamide** (**5m**). White solid; yield 52%; m.p. 108–109°C; ^1^H NMR (DMSO-*d*_6_, 600 MHz, ppm) δ: 10. 47 (s, 1H), 7.78 (s, 1H), 7.62 (d, 1H, *J* = 7.9 Hz), 7.51 (s, 1H), 7.45 (t, 1H, *J* = 8.2 Hz), 7.22–7.17 (m, 2H), 7.13 (dd, 1H, *J*_*1*_ = 1.4 Hz, *J*_*2*_ = 7.3 Hz), 7.02 (dd, 1H, *J*_*1*_ = 1.3 Hz, *J*_*2*_ = 8.0 Hz), 3.87 (s, 3H), 3.82 (s, 3H), 2.54 (s, 3H); ^13^C NMR (DMSO-*d*_6,_ 150 MHz, ppm) δ: 170.55, 169.79, 165.59, 156.31 (q, *J* = 34.8 Hz), 153.05, 152.35, 146.32, 141.05, 131.70, 130.46, 124.72, 121.85 (q, *J* = 273.45 Hz), 120.45, 117.54, 117.10, 115.23, 112.91, 103.40, 61.57, 56.42, 25.92; HRMS (calcd.) for C_21_H_18_F_3_N_3_O_4_ (M + H)^+^ 434.1322, found 434.1318.

**3-Bromo-4-chloro-*N*-(3-((2-methyl-6-(trifluoromethyl)pyrimidin-4-yl)oxy)phenyl)benzamide** (**5n**). White solid; yield 68%; m.p. 143–145°C; 1H NMR (DMSO-*d*_6_, 600 MHz, ppm) δ: 10.56 (s, 1H), 8.35 (d, 1H, *J* = 1.9 Hz), 7.97 (dd, 1H, *J*_*1*_ = 2.1 Hz, *J*_*2*_ = 8.3 Hz), 7.80 (d, 1H, *J* = 8.4 Hz), 7.76 (s, 1H), 7.68 (d, 1H, *J* = 8.1 Hz), 7.54–7.52 (m, 2H, Ph-H), 7.49–7.46 (m, 2H), 7.04 (dd, 1H, *J*_*1*_ = 1.4 Hz, *J*_*2*_ = 8.0 Hz), 2.53 (s, 3H); ^13^C NMR (DMSO-*d*_6,_ 150 MHz, ppm) δ: 170.54, 169.79, 163.71, 156.33 (q, *J* = 34.8 Hz), 152.27, 140.74, 137.07, 135.34, 133.25, 131.05, 130.42, 129.12, 122.11, 121.81 (q, *J* = 273.3 Hz), 118.31, 117.51, 113.76, 103.37, 25.92; HRMS (calcd.) for C_19_H_12_BrClF_3_N_3_O_2_ (M + H)^+^ 485.9826, found 485.9822.

**5-Bromo-2-fluoro-*N*-(3-((2-methyl-6-(trifluoromethyl)pyrimidin-4-yl)oxy)phenyl)benzamide** (**5o**). White solid; yield 52%; m.p.147–149 °C; ^1^H NMR (DMSO-*d*_6_, 600 MHz, ppm) δ: 10.74 (s, 1H), 7.89–7.87 (m, 1H), 7.79–7.77 (m, 1H, Ph-H), 7.73 (s, 1H), 7.60 (d, 1H, *J* = 7.9 Hz), 7.50 (s, 1H), 7.47 (t, 1H, *J* = 8.2 Hz), 7.38 (t, 1H, *J* = 9.3 Hz), 7.05 (dd, 1H, *J*_1_ = 1.4 Hz, *J*_2_ = 8.0 Hz), 2.53 (s, 3H); ^13^C NMR (DMSO-*d*_6,_ 150 MHz, ppm) δ: 170.52, 169.76, 161.91, 159.46, 157.80, 156.32 (q, *J* = 34.65 Hz), 152.34, 140.55, 135.67, 132.64, 130.58, 127.15, 121.83 (q, *J* = 273.15 Hz), 119.24, 117.66, 116.63, 113.19, 103.42, 25.92; HRMS(calcd.) for C_19_H_12_BrF_4_N_3_O_2_ (M + H)^+^ 470.0122, found 470.0117.

**2-Bromo-5-fluoro-*N*-(3-((2-methyl-6-(trifluoromethyl)pyrimidin-4-yl)oxy)phenyl)benzamide** (**5p**). White solid; yield 60%; m.p.152–153 °C; 1H NMR (DMSO-*d*_6_, 600 MHz, ppm) δ: 10.78 (s, 1H), 7.78 (q, 1H, *J* = 5.0 Hz), 7.72 (s, 1H), 7.59–7.58 (m, 2H), 7.52 (s, 1H), 7.48 (t, 1H, *J* = 8.2 Hz), 7.34 (dd, 1H, *J*_1_ = 3.0 Hz, *J*_2_ = 8.6 Hz, Ph-H), 7.06 (dd, 1H, *J*_1_ = 1.4 Hz, *J*_2_ = 8.0 Hz), 2.54 (s, 3H); ^13^C NMR (DMSO-*d*_6,_ 150 MHz, ppm) δ: 170.49, 169.75, 165.15, 162.38, 160.74, 156.31 (q, *J* = 34.65 Hz), 152.36, 140.82, 140.64, 135.16, 130.60, 121.84 (q, *J* = 273.15 Hz), 118.87, 117.59, 116.63, 114.21, 112.98, 103.49, 25.94; HRMS(calcd.) for C_19_H_12_BrF_4_N_3_O_2_ (M + H)^+^ 470.0122, found 470.0118.

**3,4,5-trimethoxy-*N*-(3-((2-methyl-6-(trifluoromethyl)pyrimidin-4-yl)oxy)phenyl)benzamide** (**5q**). White solid; yield 78%; m.p.116–117 °C; ^1^H NMR (DMSO-*d*_6_, 600 MHz, ppm) δ: 10.31 (s, 1H), 7.73 (s, 1H), 7.69 (d, 1H, *J* = 7.8 Hz), 7.51 (s, 1H), 7.48 (t, 1H, *J* = 7.8 Hz), 7.28 (s, 2H), 7.03 (d, 1H, *J* = 7.8 Hz), 3.87 (s, 3H), 3.74 (s, 3H), 2.51 (s, 3H); ^13^C NMR (DMSO-*d*_6,_ 150 MHz, ppm) δ: 170.59, 169.79, 165.58, 156.31 (q, *J* = 34.65 Hz), 153.11, 152.27, 141.07, 140.90, 130.37, 130.23, 120.02 (q, *J* = 272.85 Hz), 118.41, 117.19, 113.82, 105.78, 103.35, 60.58, 56.55, 25.92; HRMS(calcd.) for C_22_H_20_F_3_N_3_O_2_ (M + H)^+^ 464.1428, found 464.1425.

### *In Vitro* Antifungal Activity Test

The antifungal activities of all synthesized compounds at the concentration of 50 μg/ml were evaluated for their *in vitro* antifungal activities against the pathogenic fungi, including *B. dothidea*, *Phompsis* sp., and *B. cinerea* by the poison plate technique (Min et al., 2016). All the compounds were dissolved in 1 ml dimethyl sulfoxide (DMSO) before mixing with 90 ml potato dextrose agar (PDA). Mycelia dishes of approximately 5 mm diameter were cut from the culture medium and then picked up with a germfree inoculation needle and inoculated in the middle of the PDA plate aseptically. The inoculated plates were fostered at 27 ± 1 C for 3–4 days. DMSO in sterile distilled water served as a negative control, while Pyrimethanil acted as a positive control. For each treatment, three replicates were conducted. The inhibition rate *I* (%) was calculated by the following formula, where C (cm) represents the diameter of fungi growth on untreated PDA, and T (cm) represents the diameter of fungi on treated PDA.

I(%)=[(C−T)/(C−0.4)]×100

## Results and Discussion

### Antifungal Activity Test *in vitro*

The *in vitro* antifungal activities of the title compounds against the pathogenic fungi, including *B. dothidea*, *Phomopsis* sp., and *B. cinereal* at 50 μg/ml were tested and the results are shown in Table 1. Bioassay results showed that compounds **5i**, **5l**, **5n**, and **5o** had good inhibition rates on *B. dothidea,* with the inhibition rates of 82.1, 81.1, 84.1, and 88.5%, respectively, which were similar to Pyrimethanil (84.4%). Meanwhile, the inhibition rates of compounds **5f**, **5n**, **5o**, and **5p** against *Phomopsis* sp. were 100.0, 91.8, 100.0, and 93.4%, which were even better than Pyrimethanil (85.1%). In addition, some of the target compounds, for example compounds **5c** (80.0%), **5i** (79.6%), **5l** (80.4%), **5m** (83.6%), **5n** (79.7%), **5o** (84.7%), and **5q** (80.5%), showed equally antifungal activity against *B. cinerea* to Pyrimethanil (82.8%).

**TABLE 1:** | The antifungal activities of the title compounds against *B. dothidea*, *Phomopsis* sp*.*, and *B. cinereal* at 50 μg/ml.

Compounds	Inhibition rate (%)		
	*B. dothidea*	*Phomopsis* sp	*B. cinerea*
**5a**	75.6 ± 2.1	73.6 ± 2.2	60.3 ± 1.8
**5b**	76.2 ± 1.3	81.0 ± 1.3	73.1 ± 1.3
**5c**	62.5 ± 1.1	75.5 ± 1.8	80.0 ± 2.5
**5d**	70.5 ± 1.6	80.2 ± 2.2	70.6 ± 1.2
**5e**	54.6 ± 1.5	76.0 ± 1.4	73.3 ± 1.9
**5f**	72.3 ± 1.9	100.0 ± 2.1	59.7 ± 2.4
**5g**	46.8 ± 1.0	60.1 ± 2.0	73.8 ± 2.0
**5h**	78.5 ± 2.6	86.1 ± 1.9	75.8 ± 2.6
**5i**	82.1 ± 3.0	84.4 ± 2.1	79.6 ± 1.4
**5g**	72.4 ± 1.9	78.0 ± 2.2	72.1 ± 3.2
**5k**	76.9 ± 1.0	81.2 ± 1.8	77.5 ± 1.8
**5l**	81.1 ± 1.2	84.5 ± 1.7	80.4 ± 1.2
**5m**	48.9 ± 1.7	57.8 ± 1.3	83.6 ± 1.2
**5n**	84.1 ± 2.3	91.8 ± 1.4	79.7 ± 2.4
**5o**	88.5 ± 3.3	100.0 ± 1.0	84.7 ± 2.6
**5p**	79.9 ± 1.8	93.4 ± 1.5	75.2 ± 1.2
**5q**	54.5 ± 1.6	62.1 ± 1.4	80.5 ± 0.9
Pyrimethanil	84.4 ± 2.1	85.1 ± 1.4	82.8 ± 1.4

**FIGURE 2:**
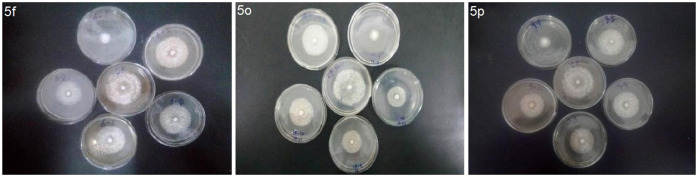
EC_50_ values of the target compounds **5f**, **5o,** and **5p** against *Phomopsis* sp.

The EC_50_ values and antifungal diagram of the target compounds **5f**, **5o**, and **5p** were also tested and are presented in Table 2 and Figure 2 respectively. Table 2 shows that compounds **5f**, **5o**, and **5p** exhibited excellent antifungal activity against *Phomopsis* sp., with the EC_50_ values of 15.1, 10.5, and 19.6 μg/ml, which were superior to that of Pyrimethanil (32.1 μg/ml).

**TABLE 2:** | The EC50 values of the target compounds against *Phompsis* sp.

Compounds	Toxic regression equation	*r*	EC_50_ (μg/mL)
**5f**	y = 2.42x + 3.62	0.99	15.1 ± 2.0
**5o**	y = 2.38x + 4.20	0.99	10.5 ± 1.4
**5p**	y = 3.45x + 2.65	0.99	19.6 ± 2.5
Pyrimethanil	y = 2.18x + 8.25	0.99	32.1 ± 2.0

### Structure-Function Relationship Analysis

In order to design novel and more promising active small molecules of pyrimidine derivatives, SAR analysis was also performed. The chemical structure of the target compounds indicated that the position of the amine group and the position and size of the substituent group R of the target compounds significantly influence the antifungal activities against*. B. dothidea*, *Phomopsis* sp. and *B. cinereal*. With the amine group at 3-position of the benzene ring and F and Br atoms at 2- and 5-position, respectively, compound **5o** exhibited excellent antifungal activities against*. B. dothidea*, *Phomopsis* sp. and *B. cinereal*, which were even better than those of Pyrimethanil. Meanwhile, in general terms, the antifungal activities of the target compounds with the amine group at 3-position of benzene ring were better than those of the corresponding target compounds with the amine group at 2-position of the benzene ring, for example, **5h** > **5b** and **5k** > **5d**.

## Conclusion

In conclusion, a total of 17 pyrimidine derivatives containing an amide moiety were synthesized and evaluated for their *in vitro* fungicidal activities against *B. dothidea*, *Phompsis* sp., and *B. cinerea* by the poison plate technique. Bioassay results demonstrated that compound 5-bromo-2-fluoro-*N*-(3-((2-methyl-6-(trifluoromethyl)pyrimidin-4-yl)oxy)phenyl)benzamide (**5o**) exhibited excellent antifungal activity against *Phompsis* sp., with the EC_50_ value of 10.5 μg/ml, which were even better than that of Pyrimethanil. This study provided a practical tool for guiding the design and synthesis of novel and more promising active small molecules of pyrimidine derivatives for controlling *Phompsis* sp., This study also demonstrated that this series of pyrimidine derivatives containing an amide moiety can be used to develop potential agrochemicals. In accordance with the pesticide registration requirements in China, further field and toxicity studies of compound **5o** will be undertaken in a future study.

## Data Availability

The original contributions presented in the study are included in the article/Supplementary Material, further inquiries can be directed to the corresponding authors.
