# Supplementary material for: Synthesis and Antifungal Activity of Pyrimidine Derivatives Containing an Amide Moiety
Source: Front Chem. 2021 Jul 12;9:695628. doi: 10.3389/fchem.2021.695628 (PMC8311460; doi:10.3389/fchem.2021.695628)
Supplement: Supplementary file 1 [file Table1.DOCX]

Supplementary Material

# intermediates 2, 3 and 4 of NMR Data

**2-methyl-6-(trifluoromethyl)pyrimidin-4-ol** (2): White [crystals](javascript:void(0);); yield 85.7%; m.p. 140-142^o^C; ^1^H NMR (DMSO-*d*_6_,600 MHz, ppm)*δ*: 13.03 (s,1H, pyrimidine-OH), 6.68 (s, 1H, pyrimidine-H), 2.36(s, 3H); ^13^C NMR (DMSO-*d*_6,_ 150 MHz, ppm)*δ*: 162.78, 162.16, 152.03, 122.33, 120.15, 111.12, 21.74.

**4-chloro-2-methyl-6-(trifluoromethyl)pyrimidine**(3): oily; yield 51.7%; ^1^H NMR (DMSO-*d*_6_,500 MHz, ppm)*δ*: 8.12 (s, 1H, pyrimidine-H), 2.68(s, 3H); ^13^C NMR (DMSO-*d*_6,_ 125 MHz, ppm)*δ*: 170.25, 162.95, 156.12, 121.64, 119.44, 116.38, 25.78.

**2-((2-Methyl-6-(trifluoromethyl)pyrimidin-4-yl)oxy)aniline (4)**: White solid; yield 62%; m.p. 145–146 °C; ^1^H NMR (DMSO-*d*_6_, 500 MHz, ppm) *δ*: 7.24(s, 1H), 6.72-6.56 (m, 3H), 6.42(t, 1H, *J* = 7.8 Hz), 5.39 (s, 2H), 2.54(s, 3H); ^13^C NMR (DMSO-*d*_6,_ 150 MHz, ppm) *δ*: 171.55, 169.85, 147.45, 142.18, 122.23, 114.96, 102.54, 25.60.

**3-((2-Methyl-6-(trifluoromethyl)pyrimidin-4-yl)oxy)aniline (4**′**)**: White solid; yield 48%; m.p. 157–158 °C; ^1^H NMR (DMSO-*d*_6_, 600 MHz, ppm) *δ*: 7.29 (s, 1H), 7.08 (t, 2H, *J* =7.8 Hz), 6.52 (d, 1H, *J* = 7.8 Hz), 6.38 (t, 1H, *J* = 2.4 Hz), 6.33 (d, 1H, *J* = 7.8 Hz), 5.38 (s, 2H), 2.54 (s, 3H); ^13^C NMR (DMSO-*d*_6,_ 150 MHz, ppm) *δ*: 171.55, 169.85, 147.45, 142.18, 122.23, 114.96, 102.54, 25.60.

# Supplementary of HRMS Figures

**Fig.S1** **5a**

**Fig.S2** **5b**

**Fig.S3** **5c**

**Fig.S4** **5d**

**Fig.S5** **5e**

 **Fig.S6** **5f**

**Fig.S7** **5g**

**Fig.S8 5h**

**Fig.S9 5i**

**Fig.S10 5j**

**Fig.S11 5k**

**Fig.S12 5l**

**Fig.S13 5m**

**Fig.S14 5n**

**Fig.S15 5o**

**Fig.S16 5p**

**Fig.S17 5q**

# Supplementary of NMR Figures


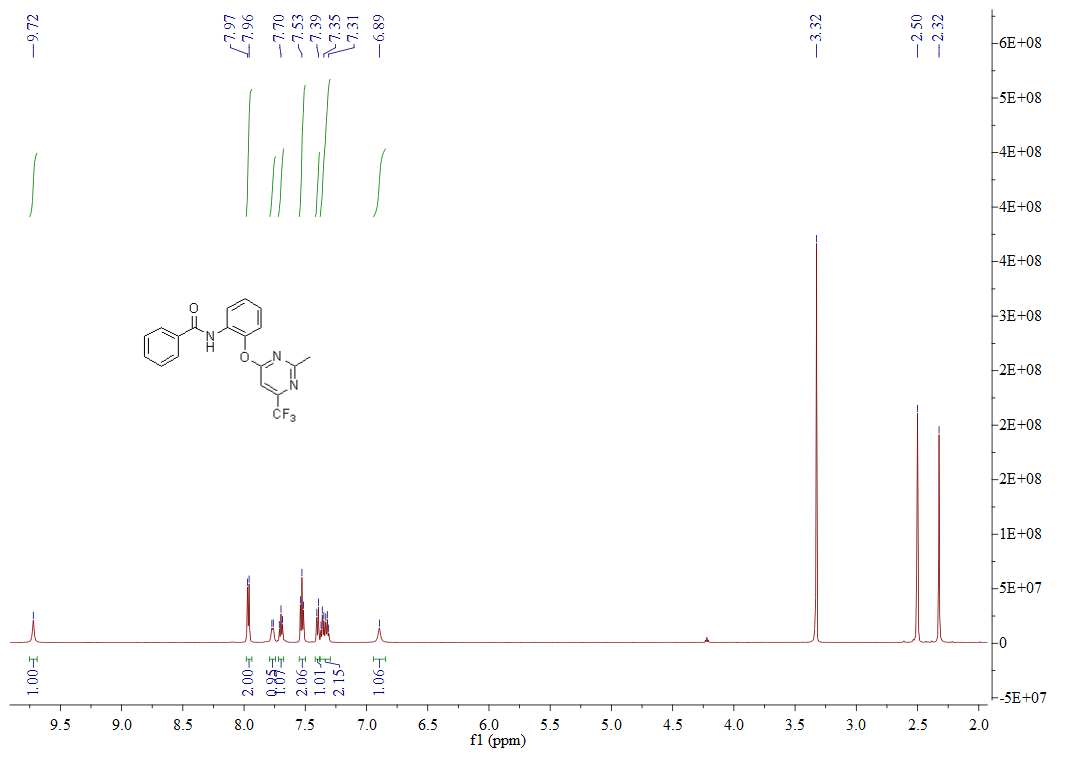


**Fig.S18 5a ^1^H NMR**


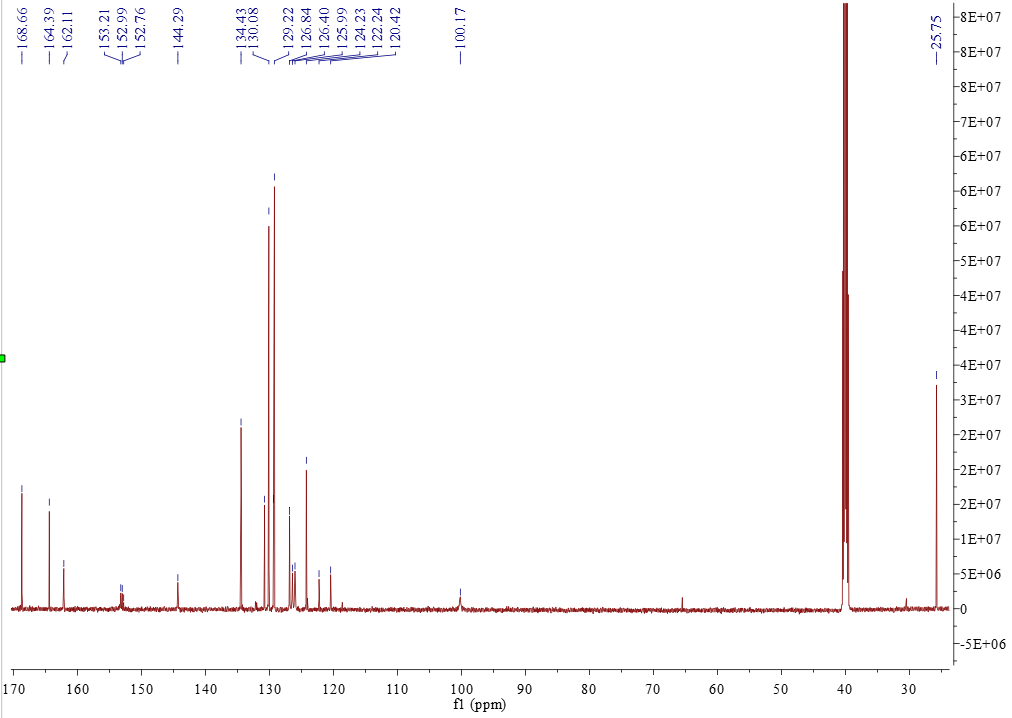


**Fig.S19 5a ^13^C NMR**


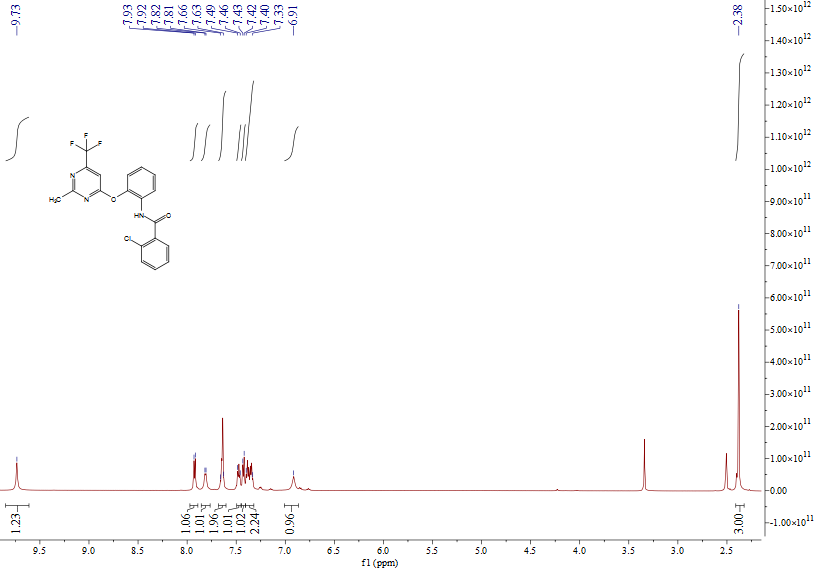


**Fig.S20 5b ^1^H NMR**


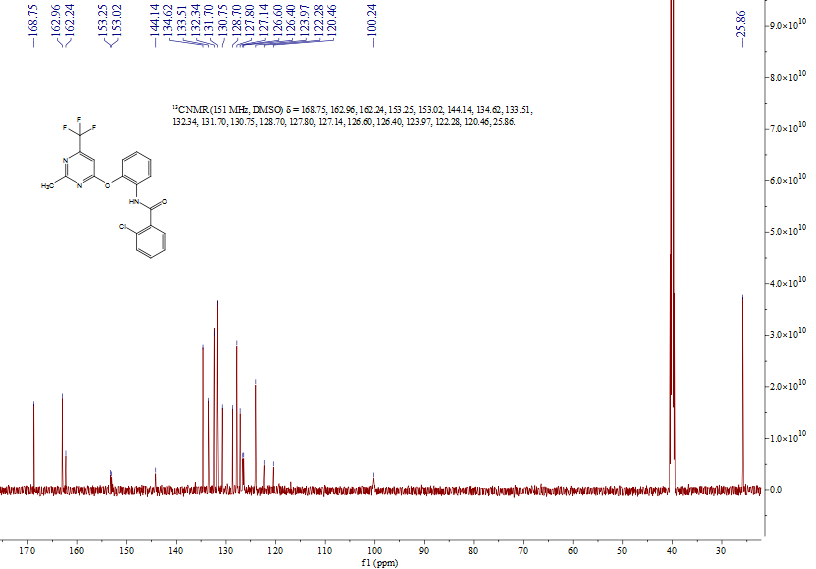


**Fig.S21 5b ^13^C NMR**


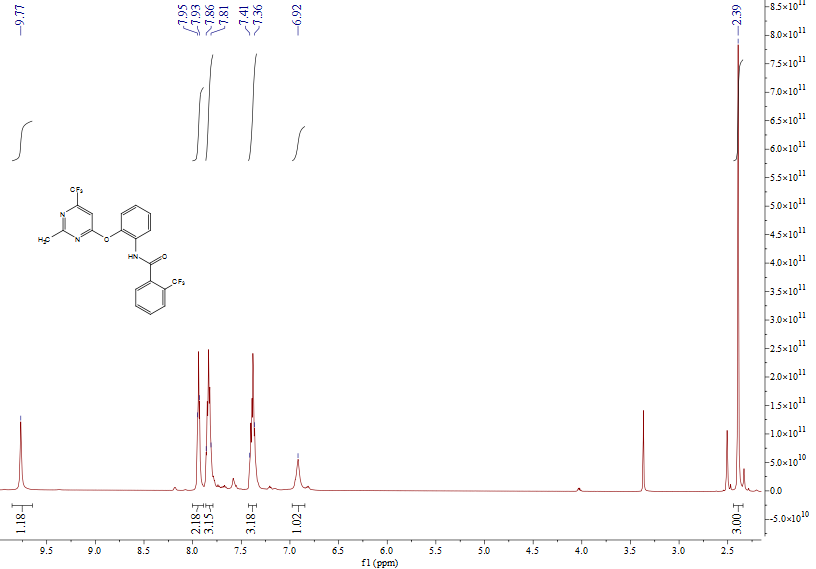


**Fig.S22 5c ^1^H NMR**


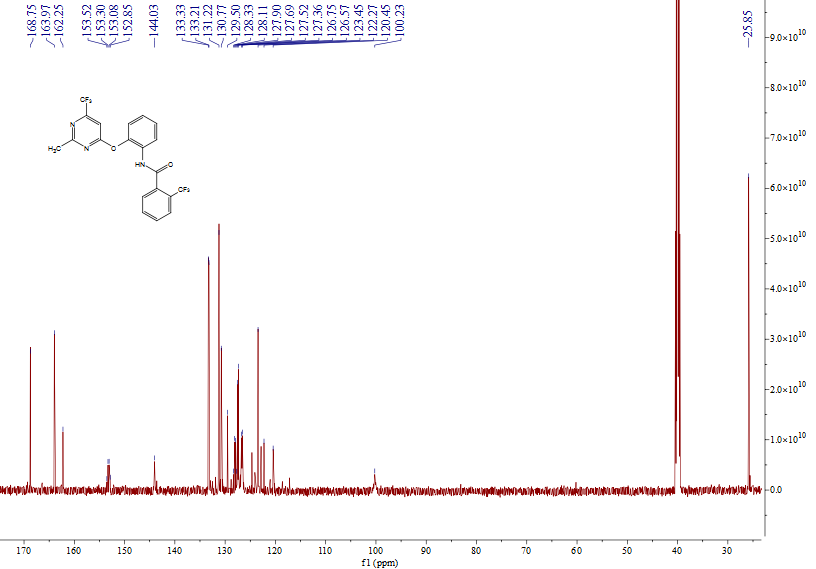


**Fig.S23 5c^13^C NMR**


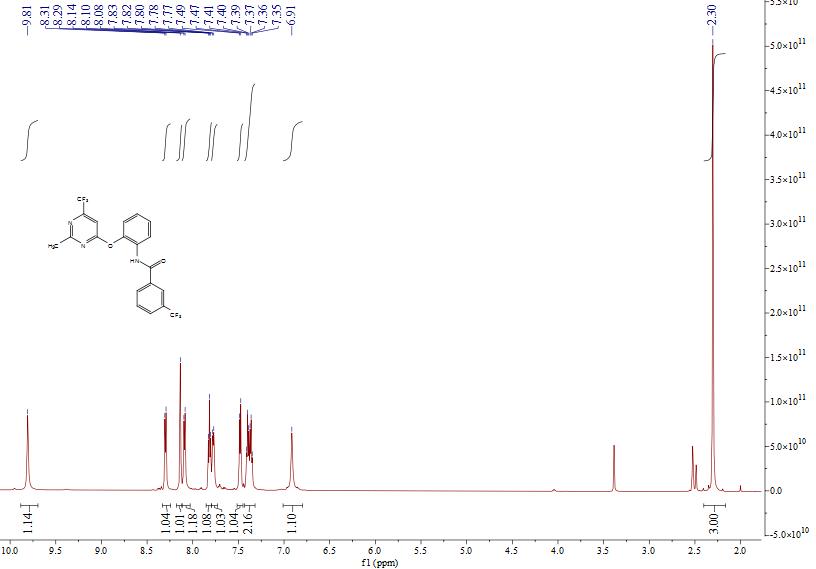


**Fig.S24 5d ^1^H NMR**


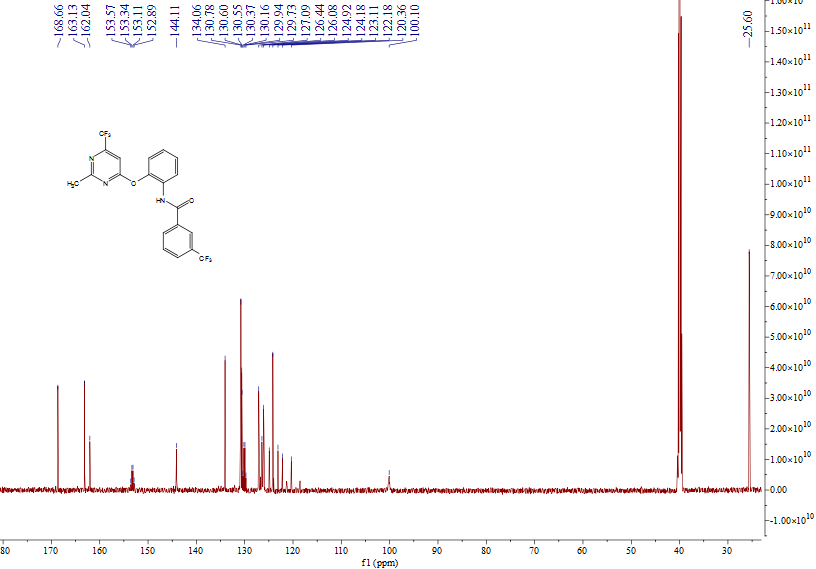


**Fig.S25 5d ^13^C NMR**


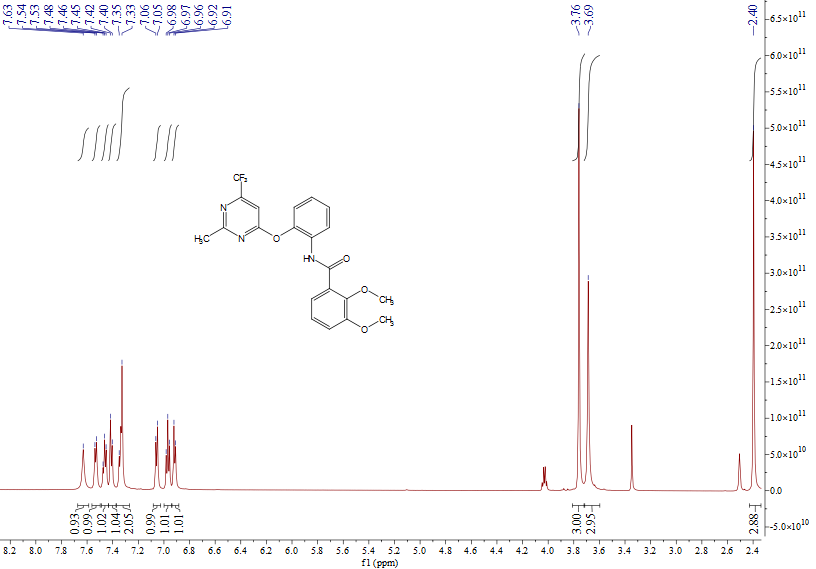


**Fig.S26 5e ^1^H NMR**


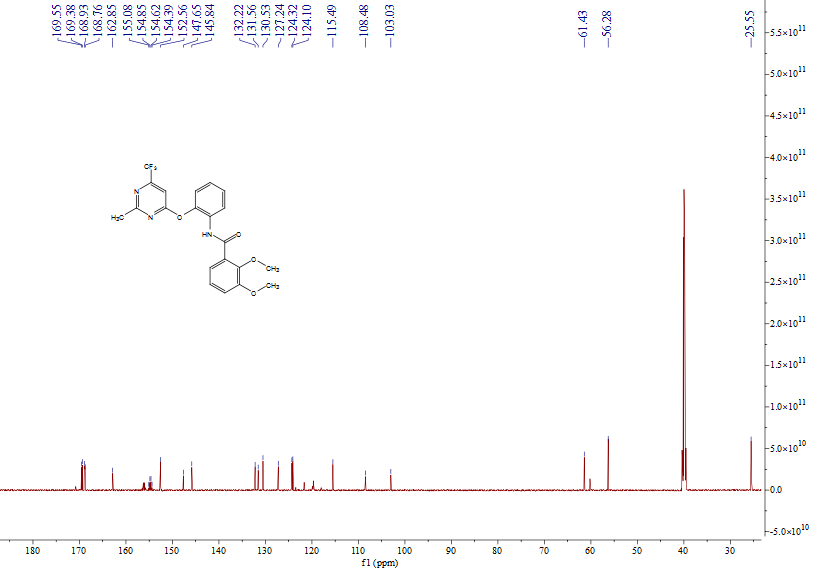


**Fig.S27 5e ^13^C NMR**


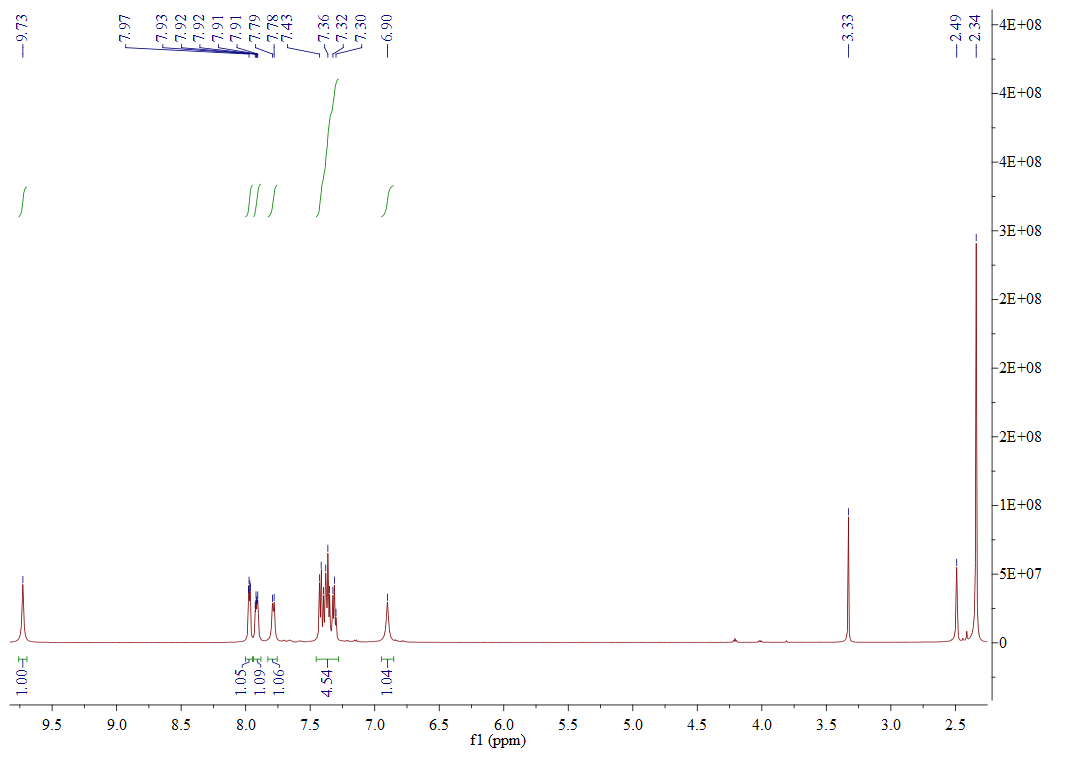


**Fig.S28 5f ^1^H NMR**


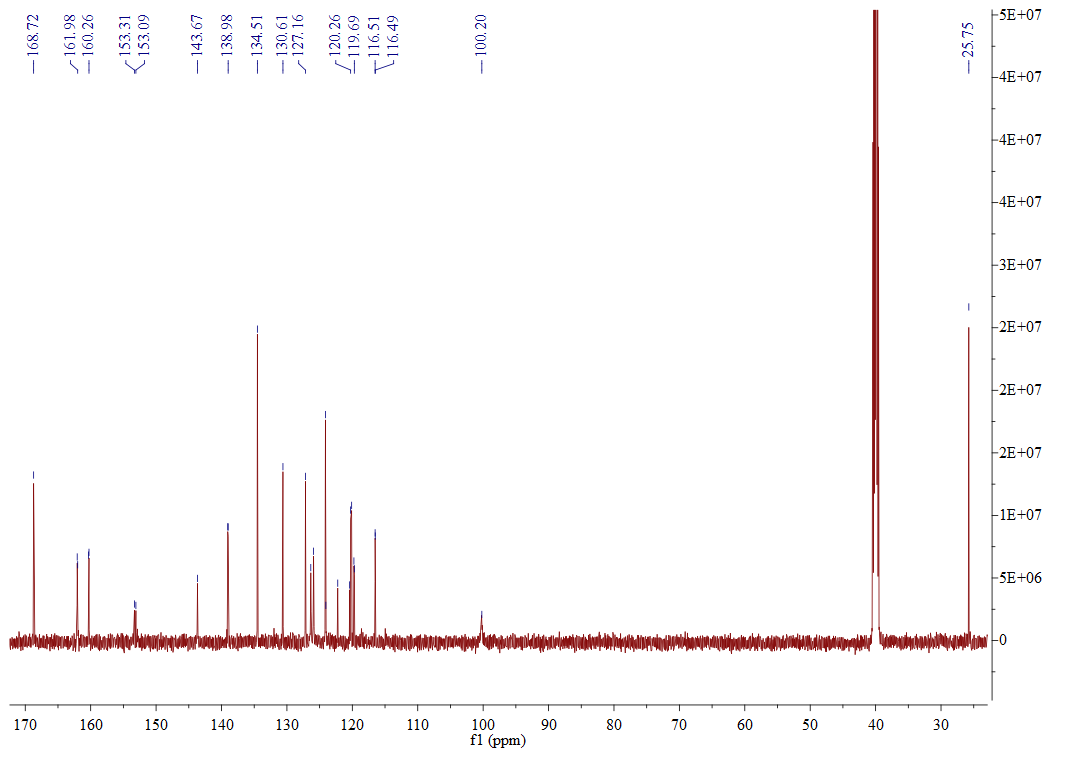


**Fig.S29 5f ^13^C NMR**


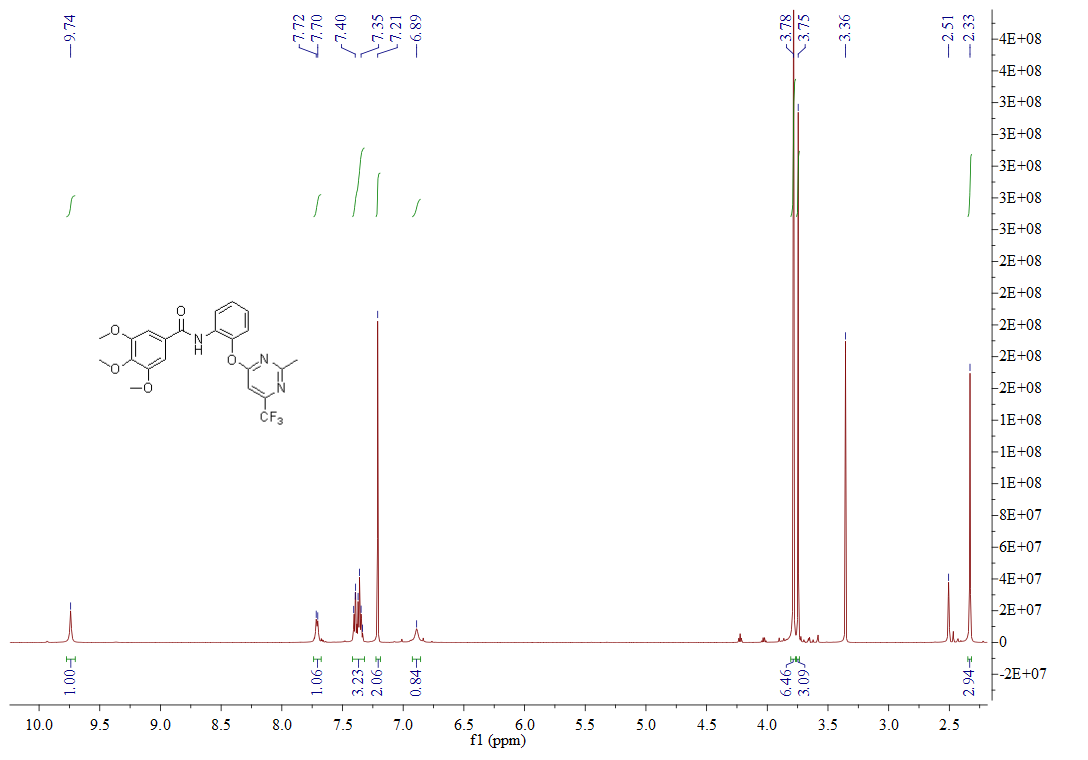


**Fig.S30 5g ^1^H NMR**


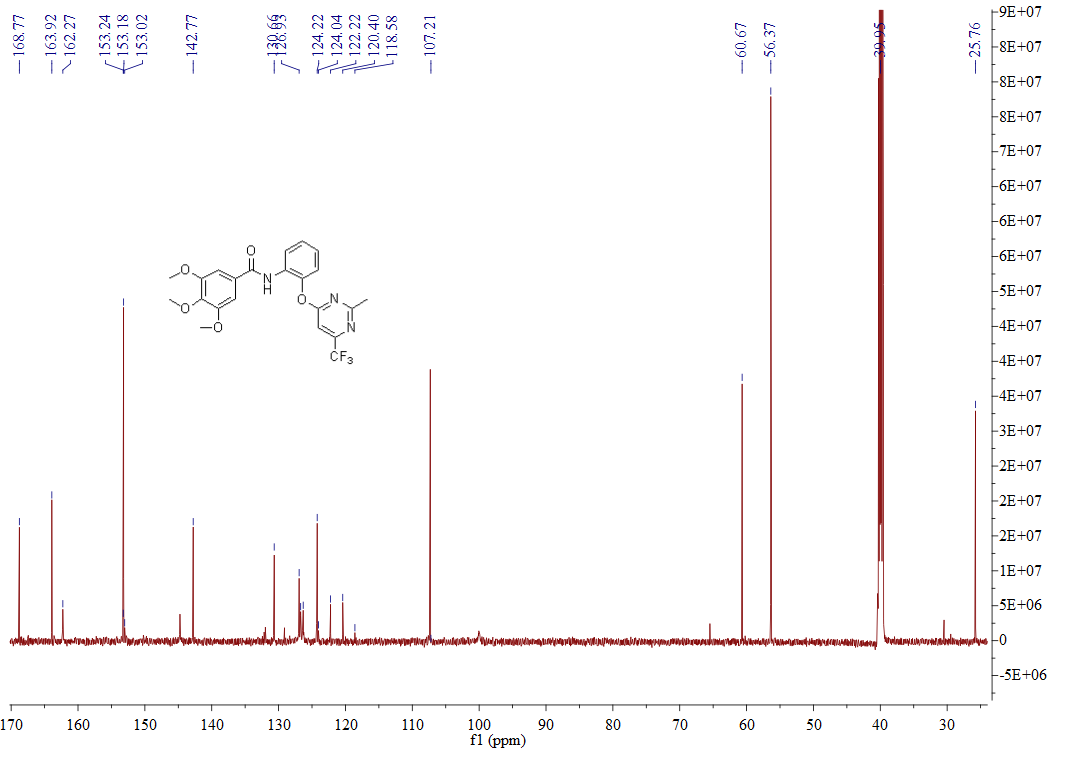


**Fig.S 31 5g ^13^C NMR**


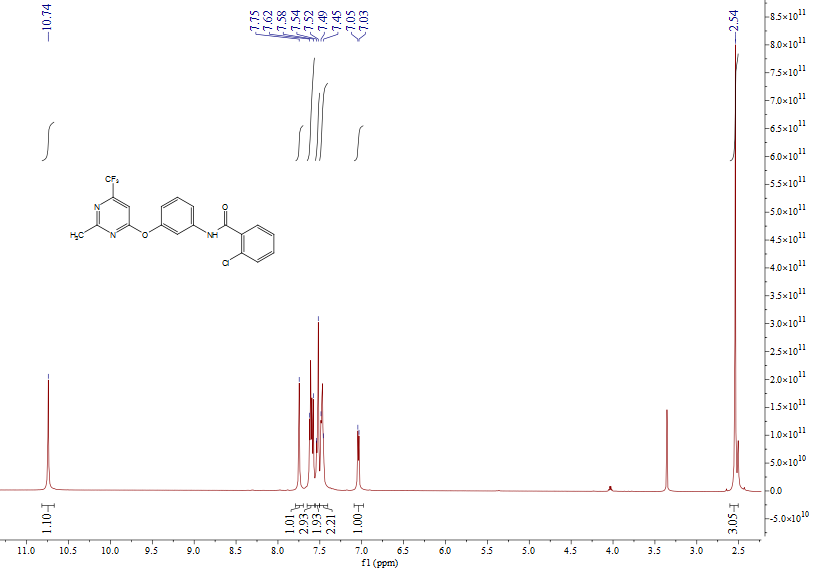


**Fig.S32 5h^1^H NMR**


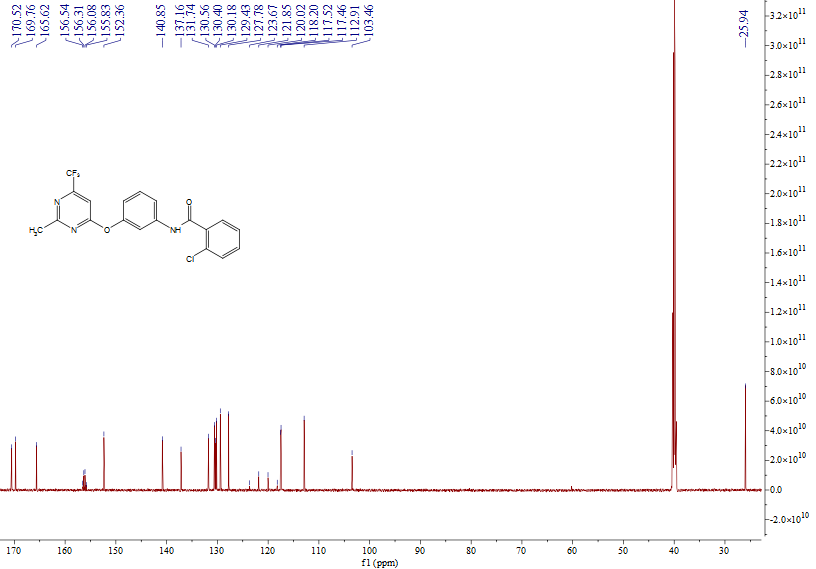


**Fig.S33 5h ^13^C NMR**


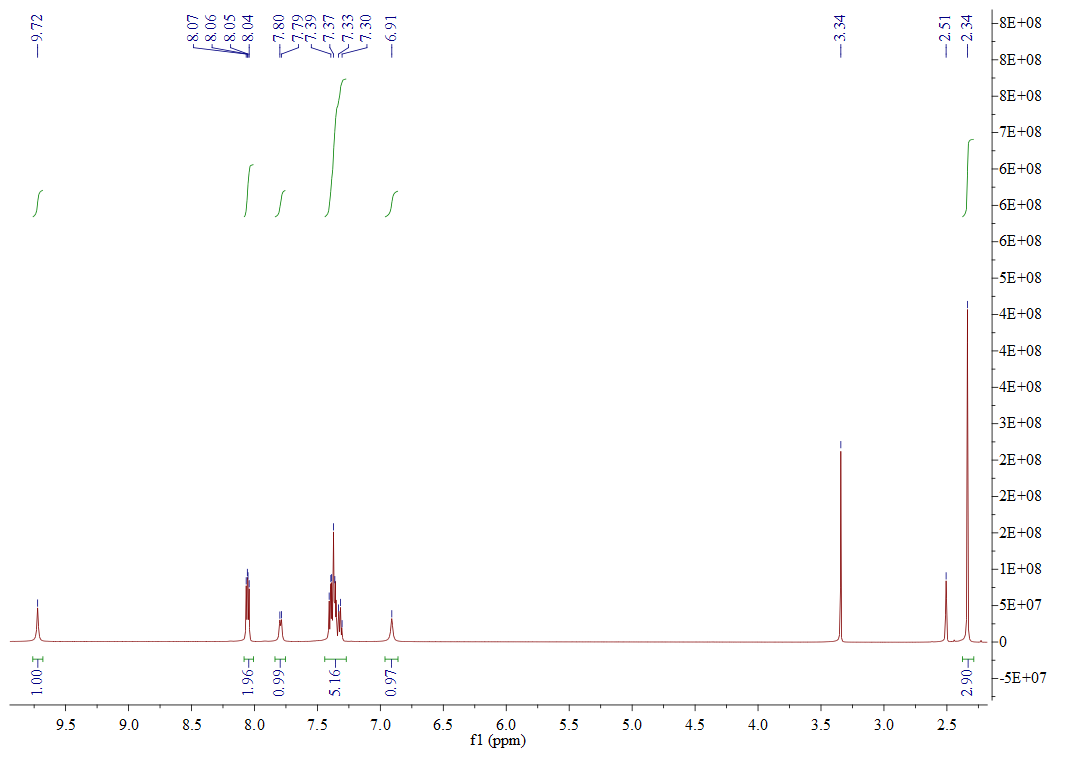


**Fig.S34 5i ^1^H NMR**


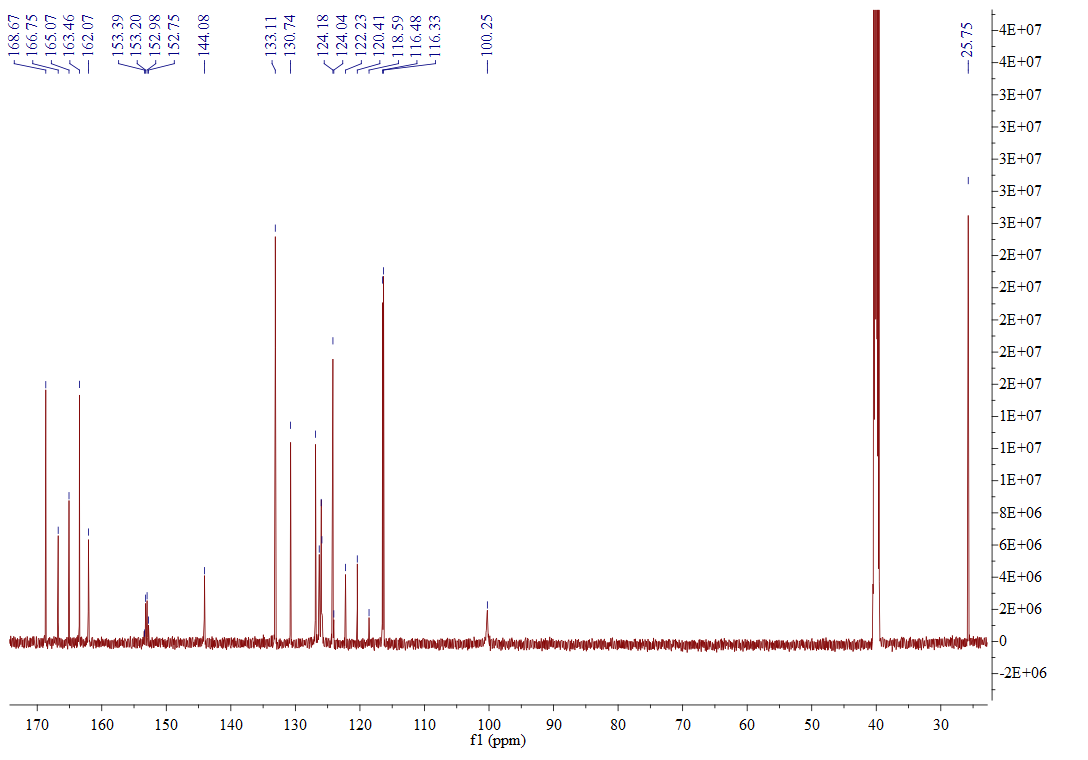


**Fig.S35 5i ^13^C NMR**


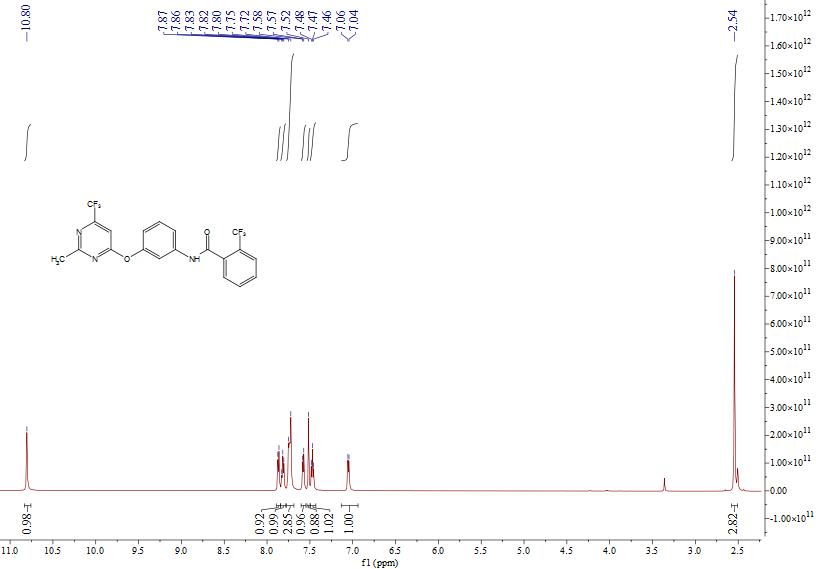


**Fig.S36 5j ^13^H NMR**


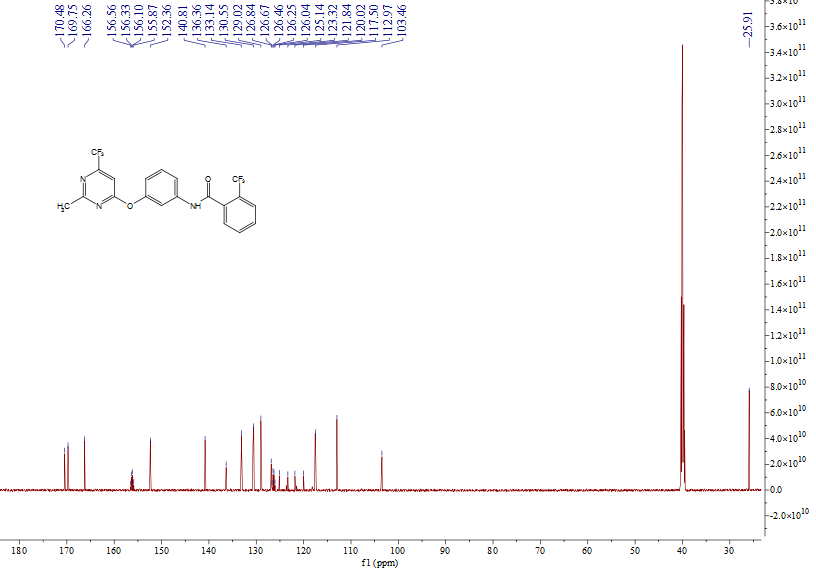


**Fig.S37 5j ^13^C NMR**


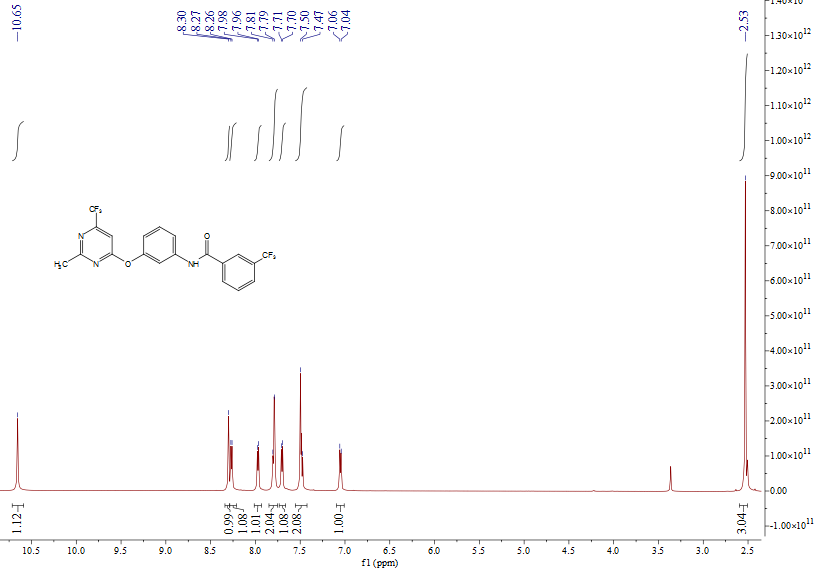


**Fig.S 38 5k ^1^H NMR**


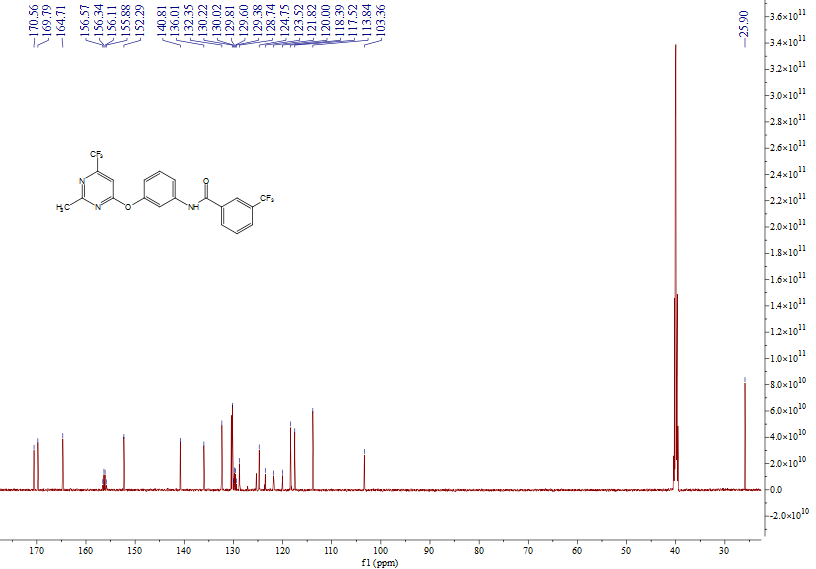


**Fig.S39 5k^13^C NMR**


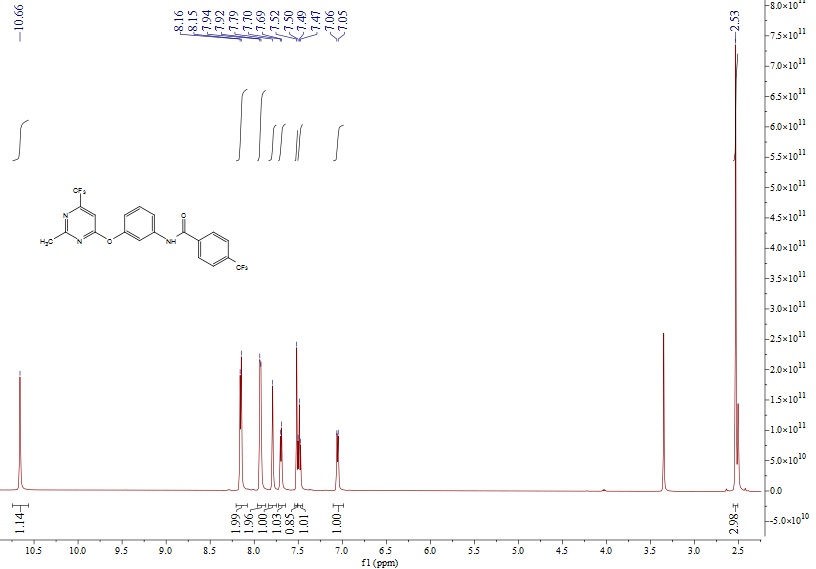


**Fig.S40 5l ^1^H NMR**


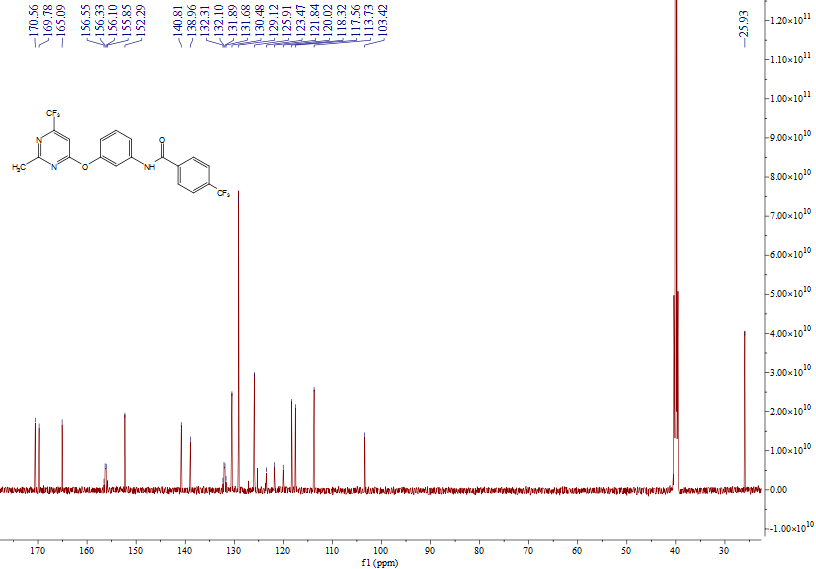


**Fig.S41 5l ^13^C NMR**


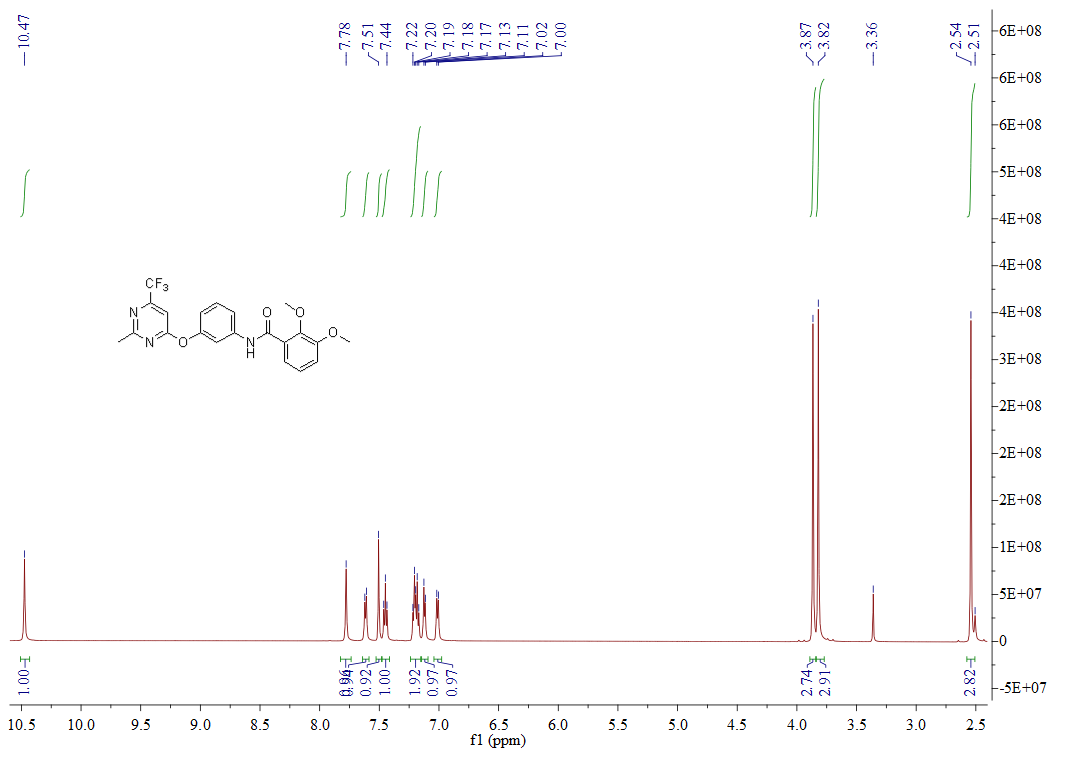


**Fig.S42 5m ^1^H NMR**


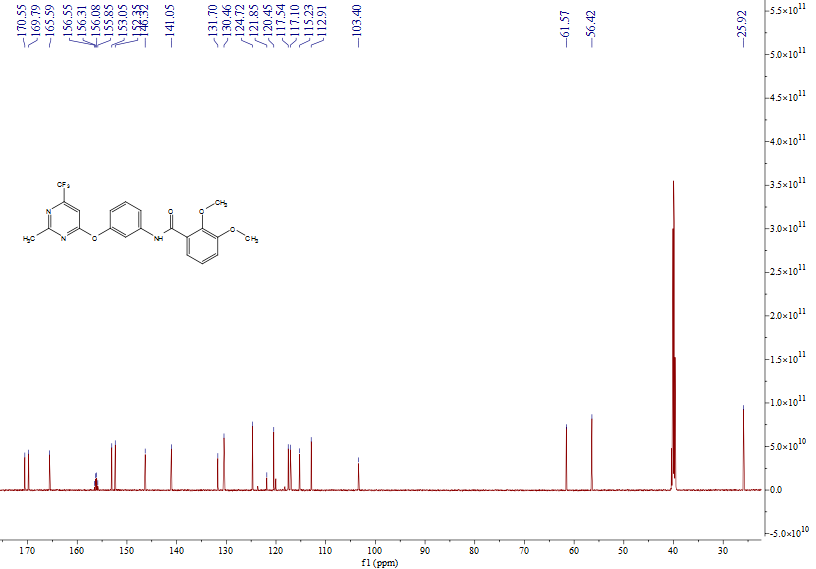


**Fig.S43 5m ^13^C NMR**


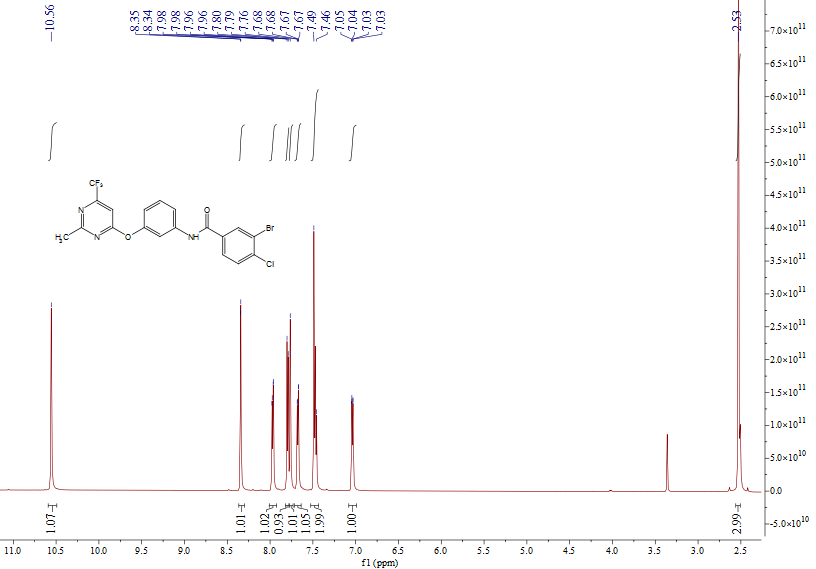


**Fig.S44 5n ^1^H NMR**


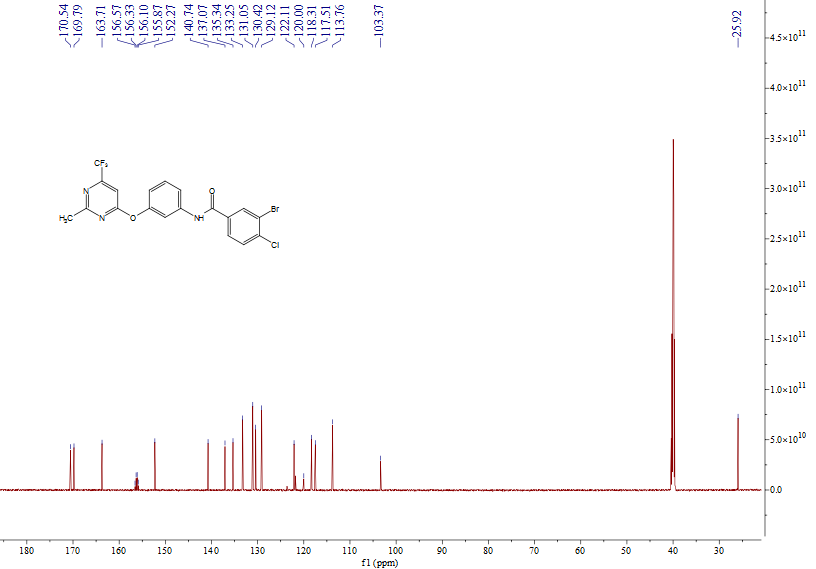


**Fig.S45 5n^13^C NMR**


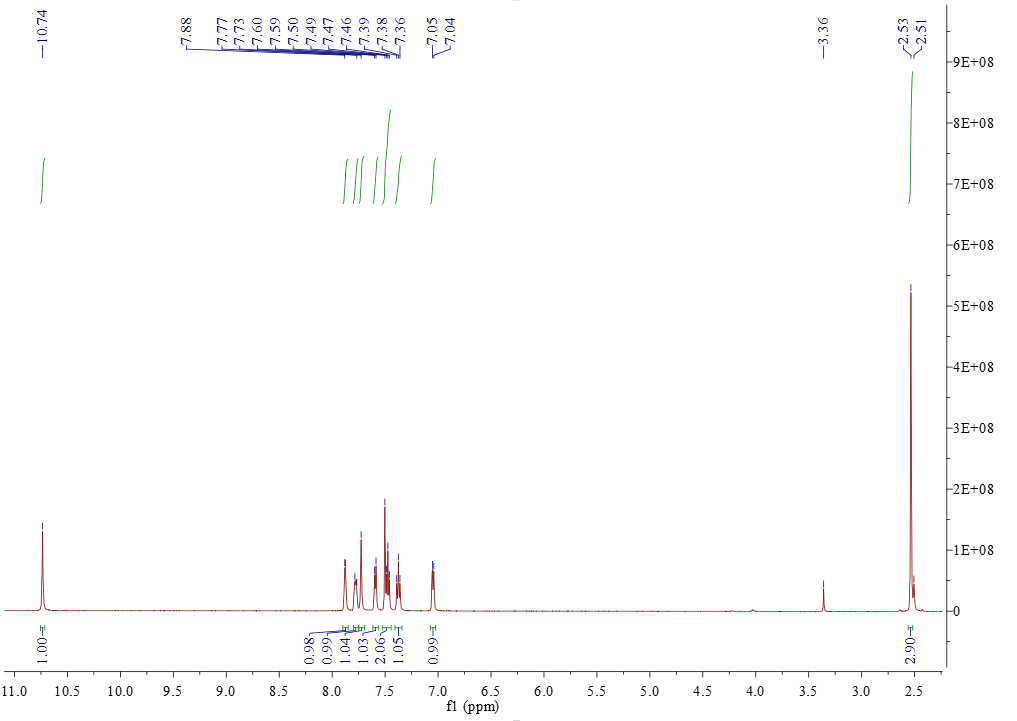


**Fig.S46 5o ^1^H NMR**


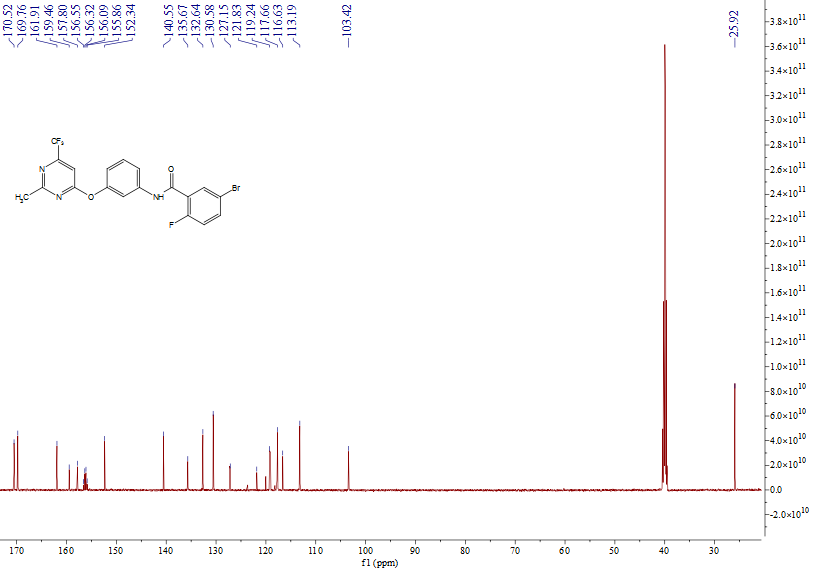


**Fig.S47 5′o ^13^C NMR**


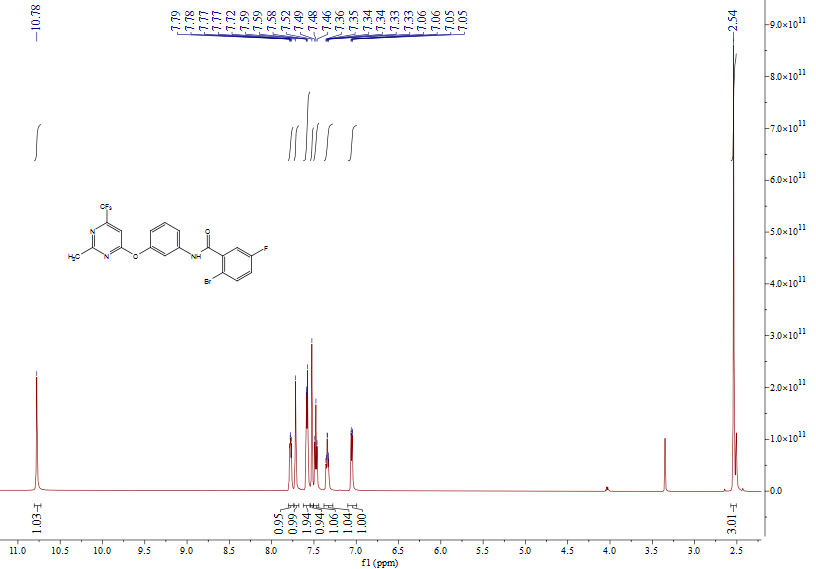


**Fig.S48 5p^1^H NMR**


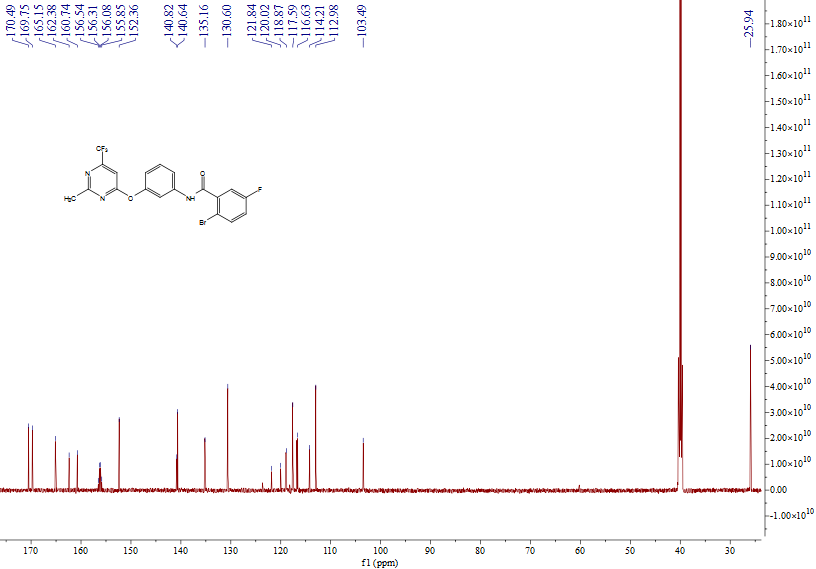


**Fig.S49 5p ^13^C NMR**


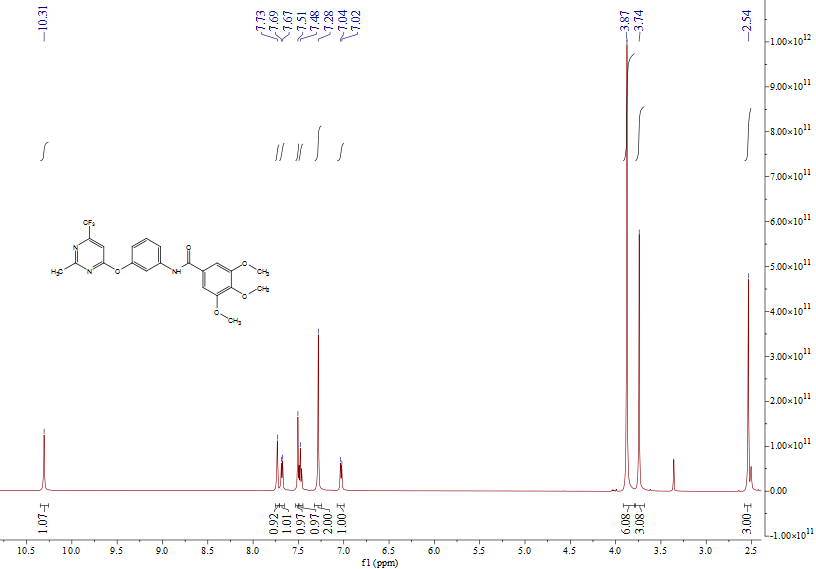


**Fig.S50 5q ^1^H NMR**


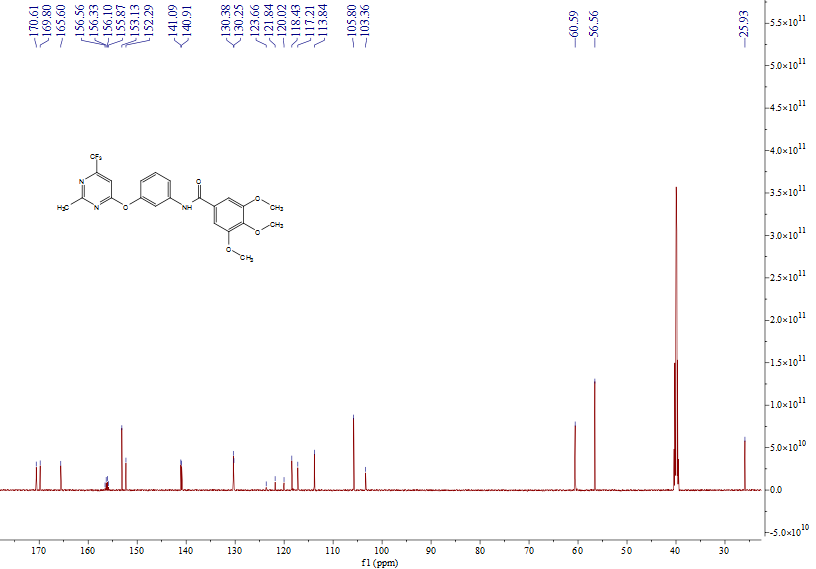


**Fig.S51 5q ^13^C NMR**
